# Pelvic gunshot wound presenting as bladder clot concealing a left external iliac injury

**DOI:** 10.1002/ccr3.7331

**Published:** 2023-05-04

**Authors:** Alain Mwamba Mukendi, Charles Eustachia Mathye

**Affiliations:** ^1^ Division of Urology, Department of Surgery, Helen Joseph Hospital University of the Witwatersrand Johannesburg South Africa

**Keywords:** arteriovesical fistula, bladder clot, bladder injury, external iliac artery injury, ilio‐vesical fistula, pelvic gunshot wound

## Abstract

**Key clinical message:**

This is the first reported case of a pelvic gunshot wound with a bladder injury masking a coinciding left external iliac artery injury. A high index of suspicion for an acute and traumatic ilio‐vesical fistula should be raised in the presence of the following triad: bright red hematuria, bladder distension from clot retention, hemodynamic instability after bladder decompression “Mukendi's triad.”

**Abstract:**

Iliac artery injury from gunshot wounds is very rare and a lethal injury associated with high mortality rate. Concurrent ballistic external iliac artery and bladder injuries resulting in an acute ilio‐vesical shunt or fistula and discovered at the time of presentation are extremely rare.

In this report, we present an unprecedented case of multiple pelvic gunshot wounds presented with a distended injured bladder full of clots concealing a left external iliac injury by tamponade effect.

## 1 INTRODUCTION

External iliac artery injuries from a penetrating pelvic trauma are rare and may result in a life‐threatening hemorrhage and death in up to 60% of cases.^1^, ^2^ These injuries are often associated with simultaneous damage to surrounding structures but rarely with bladder injury. The formation of an acute ilio‐vesical fistula or shunt as an immediate complication of both the external iliac artery and bladder injuries is extremely rare.^1^, ^2^, ^3^ Of the few reported cases of traumatic external iliac artery and bladder fistulae, only four were related to GSW. One was diagnosed at presentation and the other three occurred later from rupture of a post‐traumatic external iliac pseudoaneurysm rupturing into the bladder.^1^, ^2^, ^3^, ^4^

## 2 CASE REPORT

A 31‐year‐old male was rushed to our accident and emergency department following multiple gunshot wounds to the left scrotum and pelvis.

**FIGURE 1:**
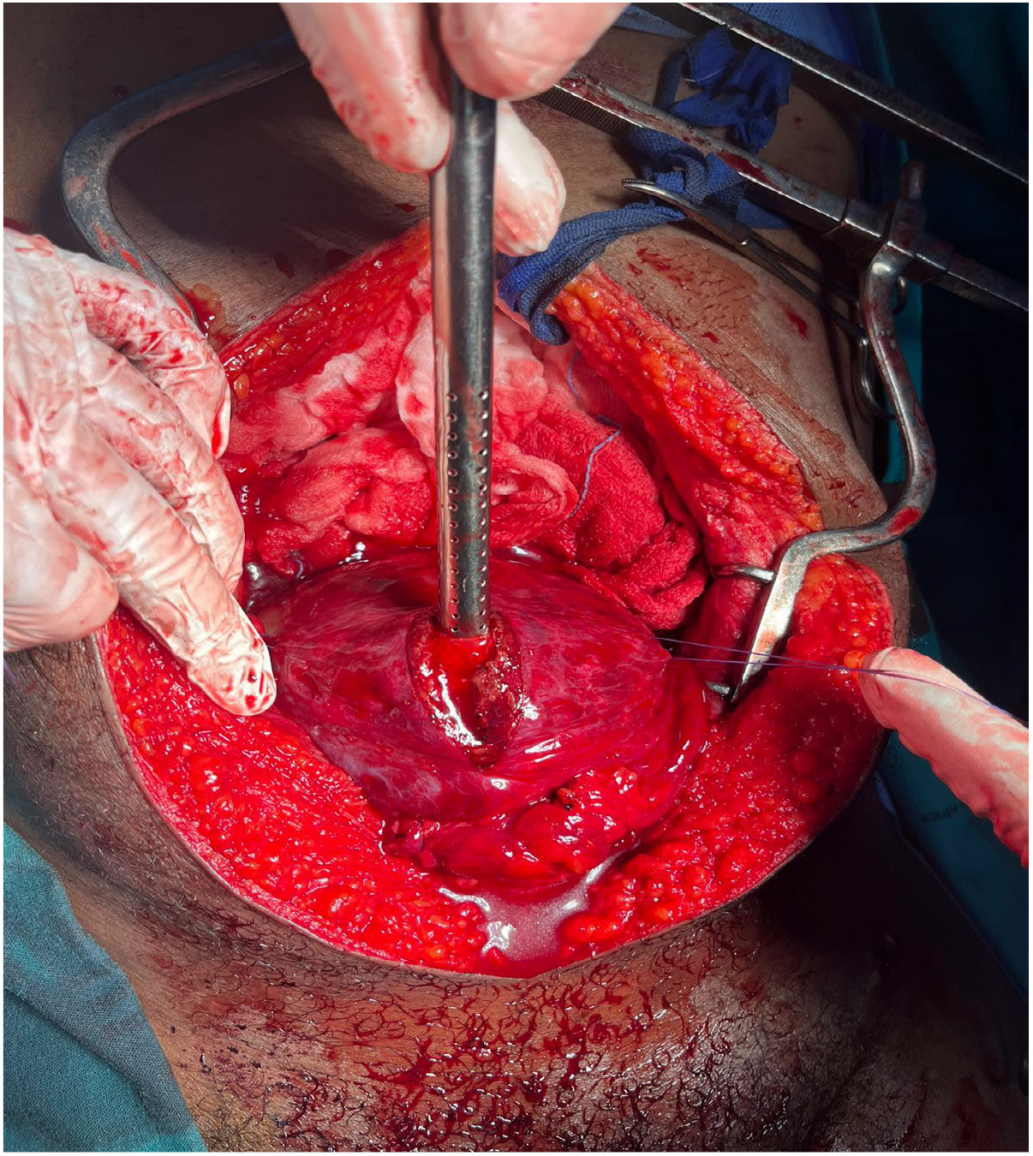
Distended erythematous bladder. A surgical incision was made to drain clots (bright red blood).

**FIGURE 2:**
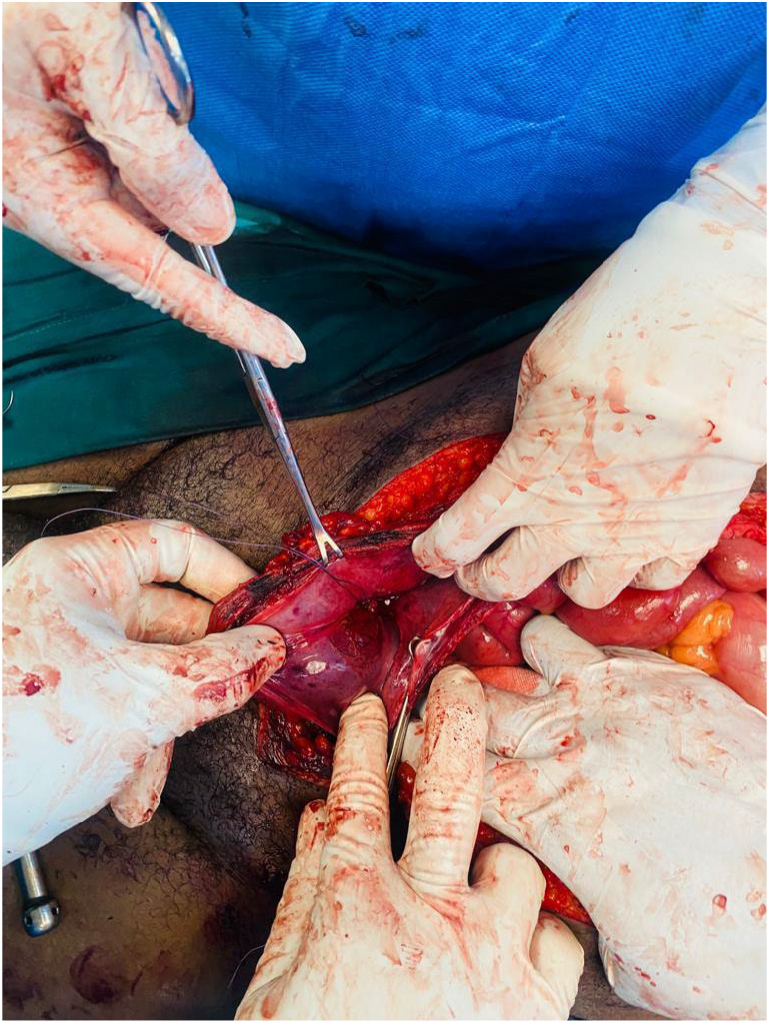
Left lateral wall defect. Through the defect a swab was used to pack the external iliac artery.

**FIGURE 3:**
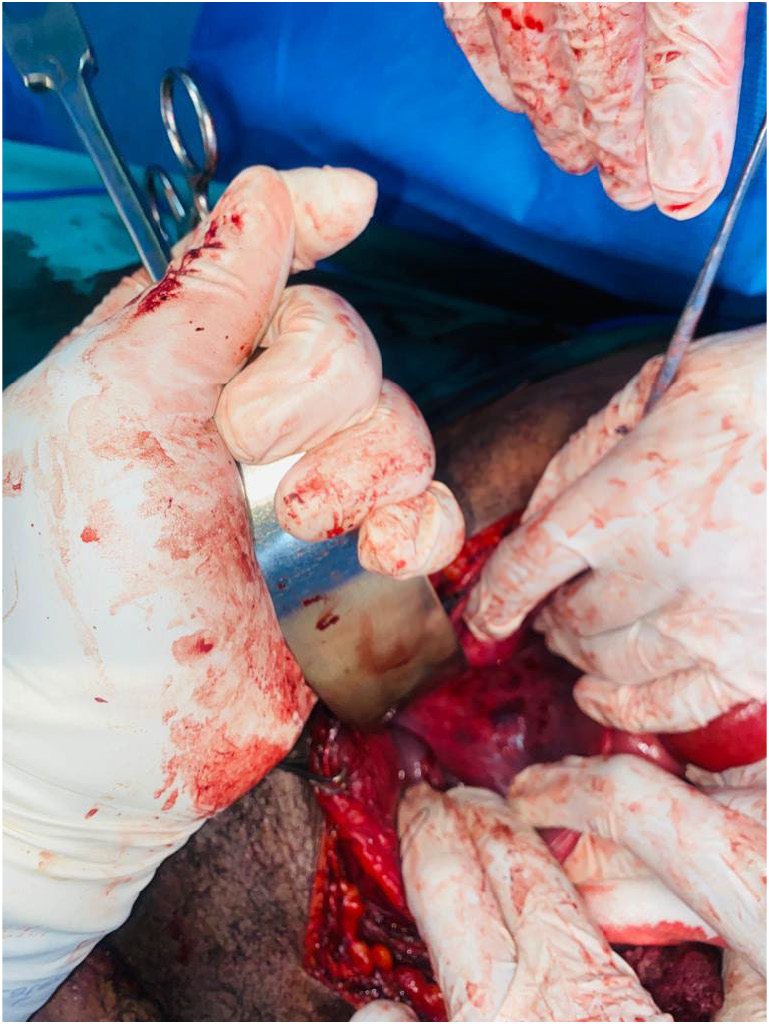
A part of the defect on the right lateral wall.

**FIGURE 4:**
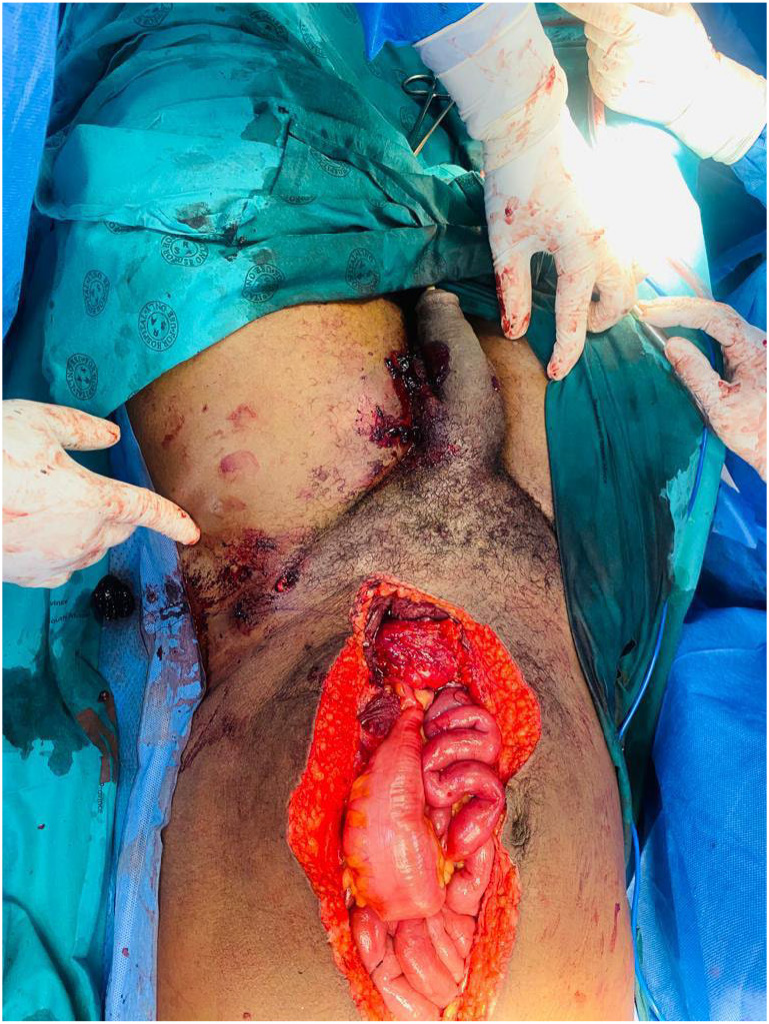
Repaired bladder and multiple pelvis and scrotal gunshot wounds.

He reported passing red bright hematuria but was hemodynamically stable with no abdominal distension, no signs of peritonitis but had mild suprapubic tenderness and a palpable bladder. We noted a through and through gunshot wound of the left hemi‐scrotum; two wounds on the left upper thigh; one on the left groin and another one on the right lower back. He had normal lower limb pulses. The focused assessment with sonography for trauma exam was negative for free fluid but showed a distended bladder. The results of hemoglobin, serum urea, electrolytes, and creatinine were within normal limits. A CT abdopelvis and angiography was done and showed bladder clot and no vascular injury. The patient was taken to the theater for an emergency scrotal exploration and cystoscopy. Scrotal exploration and left orchidectomy for shattered testis were performed followed by cystoscopy. The cystoscopy was difficult and short due to poor vision by bright red bleeding and sudden hemodynamic instability after decompressing the bladder. The procedure was immediately abandoned, and an exploratory laparotomy promptly commenced. Emergency blood was ordered, and cell saver plugged in and connected for immediate use. On entering the abdomen, there was no obvious bleeding and the patient was more stable. The bladder was noted to be distended and erythematous. A longitudinal incision was made on the anterior wall of the bladder (Figure 1) extended to the dome to evacuate the clots and identify the bleeder. A large pulsatile blood gush was noted from the left lateral wall filling the bladder with red bright blood during intravesical inspection and it was suspicious for an arteriovesical fistula. The patient became unstable again, blood transfusion was started, pulses were lost, and cardiopulmonary resuscitation (CPR) was initiated with the return of spontaneous circulation in under a minute. The bladder was packed with vascular swabs after which the Retzius space left to the bladder was inspected and bluntly dissected to expose the external iliac artery. The external iliac artery was noted to have an actively oozing laceration on its anteromedial aspect. With further dissection and exposure of the artery pulsing spurts were observed. A compression with vascular swabs was applied on it (Figure 2) and the trauma surgeon took over to repair transversally the injury with prolene sutures after gaining proximal control using a vessel loop on the left common iliac artery. All the clots in the bladder were evacuated, a 20 Fr Foley's catheter was inserted, a thorough bladder mucosa inspection done which revealed another defect on the right lateral wall of the bladder (Figure 3) away from the ureteric orifices and both ureters explored were intact. The bladder injuries were debrided and repaired, and the bladder closed in two layers (Figure 4). He demised 8 h later after another sudden hemorrhage from the same injury while in intensive care units. He became acidotic with a pH 6.8, Hb dropped to 3 from 10.7 followed by a sudden cardiac arrest. CPR commenced immediately, emergency blood ordered, and transfusion started during ongoing CPR and theater transfer preparation. There was no return of spontaneous circulation after about 20 min.

## 3 DISCUSSION

Traumatic arteriovesical fistula and specifically between the external iliac artery and the bladder is rare.^1^, ^2^, ^3^ The first reported trauma case of external iliac artery–related arteriovesical fistula was described by Rous and Andronaco^4^ following a gunshot wound to the lateral aspect of the bladder. The external iliac artery injury resulted in a pseudoaneurysm that ruptured into the bladder a week after the gunshot.^3^ Three subsequent trauma‐related cases of ilio‐vesical fistula were reported of which only one was diagnosed at presentation in an unstable patient.^1^, ^2^, ^5^ Our patient was initially hemodynamically stable, and no vascular injury was detected on CT abdomen and angiogram except a bladder clot that was noted. The patient became unstable during cystoscopy after decompressing the bladder. The arteriovesical fistula was diagnosed intraoperatively on the day of presentation during the laparotomy that ensued.

Other common causes of this entity include previous pelvic surgery and iatrogenic injury, radiotherapy, and vascular disease.^2^, ^5^

The rarity of this condition is evidenced by the paucity of literature related to it. However, in cases of recurrent and/or persistent unexplained hematuria following trauma, bladder or pelvic surgery, radiotherapy, or pelvic vascular disease, angiography can be performed to define the site, size, and extent of the fistula.^2^ In our case, hematuria was also present, but the angiography did not reveal any vascular injuries. The injury was concealed by the bladder distended by clots from the left external iliac artery bleeding into the bladder.

Interestingly, we noted three signs which were linked to this acute arteriovesical fistula, including bright red hematuria, clot retention or distended bladder, and hemodynamic instability upon disobstruction or catheterization. In a setting of penetrating pelvic trauma, urologists and/or trauma surgeons should have a high index of suspicion for an acute and traumatic arteriovesical fistula in the presence of this triad.

Once the diagnosis is made, a therapeutic plan should promptly be put in place. There is no agreement or guidelines in the literature with regard to managing this entity. However, in the emergency setting and when the patient is unstable, an open surgical approach and repair of the defects is the more likely option as there is a need to evaluate for other injuries.^2^, ^3^ In a delayed diagnosis with pseudoaneurysm, open options include repair, resection of the aneurysm, or endovascular option such as embolization.^2^, ^3^ This particular case was managed with an open repair after proximal control at the level of the left common iliac artery.

## 4 CONCLUSION

External iliac artery injuries are rare and should be promptly recognized and urgently managed to reduce the already known high mortality rate. Acute traumatic ilio‐vesical fistula is very uncommon, and this report was the first to describe a case of bladder injury concealing a concurrent external iliac artery injury.

In a setting of penetrating pelvic trauma, urologists and/or trauma surgeons should have a high index of suspicion for an acute and traumatic arteriovesical fistula or shunt in the presence of the following triad: bright red hematuria, clot retention or palpable bladder, hemodynamic instability after bladder decompression “Mukendi's triad”.

## AUTHOR CONTRIBUTIONS

**Alain Mwamba Mukendi:** Conceptualization; data curation; resources; writing – original draft; writing – review and editing. **Charles Eustachia Mathye:** Writing – original draft.

## FUNDING INFORMATION

None.

## CONFLICT OF INTEREST STATEMENT

The authors declare no conflicts of interest.

## ETHICAL APPROVAL

The study was approved by the University of the Witwatersrand Human Research Ethics Committee (HREC Medical; R14/49, Certificate number: M230389). All procedures performed in studies involving human participants were in accordance with the ethical standards of the institutional and/or national research committee and with the 1964 Helsinki Declaration and its later amendments or comparable ethical standards. Patient informed consent was obtained for publication and is available on request.

## CONSENT

Written informed consent was obtained from the Head of Department Dr. Mathye for publication of this manuscript and accompanying pictures as the patient was unconscious until demised and we could not trace any family member. A copy of the written consent is available for review by the Editor‐in‐Chief of this journal.

## Data Availability

The data that support the findings of this study are available from the corresponding author upon reasonable request.
